# Nonpeghylated liposomal doxorubicin combination regimen (R‐COMP) for the treatment of lymphoma patients with advanced age or cardiac comorbidity

**DOI:** 10.1002/hon.2764

**Published:** 2020-07-09

**Authors:** Luigi Rigacci, Ombretta Annibali, Sofya Kovalchuk, Elisabetta Bonifacio, Francesca Pregnolato, Francesco Angrilli, Umberto Vitolo, Samantha Pozzi, Serena Broggi, Stefano Luminari, Francesco Merli, Michele Spina, Silvia Bolis, Gloria Margiotta‐Casaluci, Rosario Scalzulli, Christina Cox, Angela Maria Mamusa, Armando Santoro, Pier Luigi Zinzani, Samantha Ferrari, Guido Gini, Maria Luigia Vigliotti, Antonino Mulè, Leonardo Flenghi, Fondazione Italiana Linfomi (FIL)

**Affiliations:** ^1^ UOC Ematologia e Centro Trapianto Cellule Staminali, AO San Camillo Forlanini Roma Roma Italy; ^2^ SOD C Ematologia, AOU Careggi Firenze Italy; ^3^ Ematologia, Trapianto Cellule Staminali, Medicina Trasfusionale, Policlinico Universitario Campus Biomedico Roma Italy; ^4^ Ematologia e Trapianto Midollo Osseo, Ospedale Santa Maria della Misericordia, Azienda Ospedaliera Perugia Perugia Italy; ^5^ Istituto Auxologico Italiano (IRCCS) Experimental Laboratory of Immunorheumatology, Cusano Milanino Milanino Italy; ^6^ Unità Operativa Semplice Dipartimentale Centro Diagnosi e Terapia Linfomi, Presidio Ospedaliero Pescara Italy; ^7^ SC di Ematologia, AOU Città della Salute e delle Scienze di Torino Torino Italy; ^8^ Dipartimento Onco‐ematologico, Policlinico di Modena Univesrità di Modena e Reggio Emilia Modena Italy; ^9^ Unità Operativa di Ematologia, Arcispedale S. Maria Nuova Reggio Emilia Emilia Italy; ^10^ Centro di Riferimento Oncologico di Aviano IRCCS, Aviano Italy; ^11^ Ematologia, Ospedale San Gerardo Monza Monza Italy; ^12^ Ematologia Azienda Ospedaliera Universitaria Maggiore della Carità Novara Novara Italy; ^13^ Ematologia Casa Sollievo della Sofferenza San Giovanni Rotondo Italy; ^14^ Ematologia, Ospedale Sant'Andrea Roma Roma Italy; ^15^ Ematologia e Centro Trapianto Midollo Osseo, Ospedale Businco Cagliari Cagliari Italy; ^16^ Department of Biomedical Sciences Milano Ematologia, Humanitas Clinical and Research Center – IRCCS, Humanitas University Rozzano Italy; ^17^ Institute of Hematology L.A Seragnoli, Ospedale Bologna Bologna Italy; ^18^ Unità Operativa di Ematologia, Spedali Civili di Brescia Brescia Italy; ^19^ SOD Clinca Ematologica, AOU Ospedali Riuniti Ancona Ancona Italy; ^20^ Ematologia Azienda Ospedaliera Sant'Anna e San Sebastiano Caserta Italy; ^21^ UOC Ematologia e Talassemia PO Sant'Elia Caltanissetta Caltanissetta Italy

**Keywords:** advanced age, cardiopathy, cardiotoxicity, chemotherapy, liposomal doxorubicin, lymphoma

## Abstract

Doxorubicin is the most effective single agent in the treatment of non‐Hodgkin's lymphoma (NHL). Its use is limited because of the cardiac toxicity primarily in elderly patients (pts) and in pts with history of cardiac disease. Liposomal doxorubicin has been proven to reduce cardiotoxicity. The aim of this retrospective study was the use of nonpeghylated liposomal doxorubicin (NPLD) in term of efficacy, response rate and incidence of cardiac events. We retrospectively collected the experience of 33 Hematological Italian Centers in using NPLD. Nine hundred and forty‐six consecutive pts treated with R‐COMP (doxorubicin was substituted with NPLD, Myocet) were collected. Median age was 74 years, the reasons for use of NPLD were: age (466 pts), cardiac disease (298 pts), uncontrolled hypertension (126 pts), other reasons (56 pts). According to clinicians' evaluation, 49.9% of pts would not have used standard doxorubicin for different situations (age, cardiomyopathy, previous use of doxorubicin, and uncontrolled hypertension). Overall 687 pts (72.6%) obtained a complete remission (CR). About 5% (n = 51) of subjects developed major cardiotoxic events including heart failure (N = 31), ischemic heart disease (N = 16), acute heart attack (N = 3), and acute pulmonary oedema (N = 1). After a median follow‐up of 32 months, 651 pts were alive and the overall survival (OS) was 72%. After a median observation period of 23 months disease free survival (DFS) was 58%. Either in univariate or in multivariate analysis OS and DFS were not significantly affected by age or cardiac disease. Our findings strongly support that including R‐COMP is effective and safe when the population is at high risk of cardiac events and negatively selected. Moreover, the use of this NPLD permitted that about half of our population had the opportunity to receive the best available treatment.

## 1 INTRODUCTION

Anthracyclines play a key role in the treatment of lymphoproliferative disorders and its introduction since 1970^1^ in CHOP regimen led to very important results in the cure of lymphomas. Particularly in diffuse large B cell lymphoma (DLBCL) CHOP regimen has confirmed its superiority over many other schemes used.^2^ The combination of monoclonal anti‐CD20 antibody (rituximab) with CHOP has significantly improved the efficacy of the regimen and R‐CHOP is nowadays the standard therapy for DLBCL.^3^ Unfortunately, anthracyclines are associated with cardiotoxicity, mainly congestive heart failure (CHF), that can occur during treatment (acute toxicity) or several years after treatment.^4^ Anthracycline toxicity is dose dependent and a cumulative dose of doxorubicin of 550 mg/m^2^ is associated with 30% risk of cardiovascular disease but CHF or other cardiotoxicities are now known to occur at a lower cumulative dose of 200 mg/m^2^. Using treatment without anthracyclines to mitigate the cardiovascular risk is associated to a poorer disease control and worse survival^5^ suggesting that it is not possible to propose a first line therapy without anthracyclines when the objective of the treatment is cure. Different approaches were used to avoid the cardiotoxic risk such as limiting anthracyclines dosage but this procedure could be reasonable in very elderly patients to reduce the whole toxicity^6^, ^7^ whereas is often associated with a worse outcome in younger population. Drugs as for example dexrazoxane, have been demonstrated their protective effect in pediatric but not in adult population.^8^, ^9^, ^10^ The use of lyposomal non peghilated anthracyclines (Myocet) firstly used in the treatment of patients affected by breast cancer showed in clinical studies a reduction of cardiotoxicity.^11^, ^12^ This drug was subsequently used in the treatment of aggressive lymphoma substituting hydroxydaunorubicin in the CHOP schema (called COMP). Several studies in Hematology setting confirmed in high risk and highly selected population the efficacy and particularly the safety of the COMP Scheme.^13^, ^14^, ^15^, ^16^, ^17^, ^18^ Due to a large territorial experience in Italy with this drug, thanks to the possibility to use it according to Italian law 648/96, we retrospectively collected clinical information from a large number of Hematological Italian Centers with the aim to evaluate the effectiveness and safety of this regimen in collaboration with the Fondazione Italiana Linfomi.

## 2 PATIENTS AND METHODS

According to Italian law 648/96 and starting from 2008 in Italy liposomal nonpeghilated anthracyclines could be used in elderly patients (over 65 years) or in cardiopathic patients. Then we had the opportunity to collect a large amount of data from several Hematological Italian Centers, including patients treated with this type of anthracycline within the classical regimen R‐CHOP.

Data were collected in a centralized data set from medical records of the single Institutions collecting: some essential clinical characteristics, response to therapy, overall survival and event free survival, cardiologic events observed during therapy and during the first 2 years of follow‐up. Moreover, we wanted to register why patients were treated with liposomal anthracycline, to know if the clinicians should have used standard anthracycline or they have declined the use of anthracycline.

The primary end point was the number of cardiologic complications. Secondary end points were response to therapy, overall survival, disease free survival, and event free survival.

### 2.1 Efficacy and safety response

Although a group of patients was evaluated with FDG‐PET, responses to therapy (complete response: CR; partial response: PR; progressive disease: PD; non responders: NR) were defined according to 1999 Cheson et al criteria.^19^ Overall response rate (ORR) was calculated as the sum of complete and partial response rate. Disease‐free survival (DFS) was measured from the end of the therapy to relapse or last contact, and the curves were determined using only patients in CR after induction therapy. Overall survival (OS) was dated from the disease onset to last contact. Event‐free survival (EFS) was assessed for major cardiac events (heart failure, ischemic heart disease, acute heart attack, and acute pulmonary oedema) and was dated from the end of the therapy. If a cardiac event was observed during therapy, the treatment was stopped and the time point registered as the end of therapy date.

Safety response and all cardiac events were defined according to the National Cancer Institute's Common Toxicity Criteria.^20^

### 2.2 Statistical analysis

Descriptive statistics was used to summarize continuous and categorical variables.

The Kaplan‐Meier method was used to estimate EFS, DFS, and OS. The log rank test was used to compare the survival curves of groups that were defined according to different prognostic factors. A competing risk analysis was carried out with death from cardiotoxicity as the primary event of interest and death from all other causes as competing event.

Cox regression models were used to investigate the predictors of mortality from all causes and the predictors of relapse. The predictive and confounders factors included both demographics (gender, age) and clinical characteristics (IPI class, symptoms (yes/no), year of diagnosis, previous cardiac disease (yes/no), LVEF (normal/abnormal), number of R‐COMP cycles (≤3/>3), RT (yes/no), concomitant therapies as cardiotoxic or cardioprotective drugs (yes/no). Stratification was applied in the Cox regression model when the proportional hazard assumption was violated.

The association of CR and PR with demographic and clinical patients' characteristics was assessed by performing a multinomial logistic regression since the proportional odds assumption underlying the ordinal logistic model was violated.

A *P*‐value <.05 was considered as statistically significant. All statistical analyses were performed using the R 3.6.0 for Windows.^21^

## 3 RESULTS

### 3.1 Characteristics of the study population

Data from 946 patients treated from 1999 and 2015 with R‐COMP were collected from 33 Italian Hematological Centers. The median number of cases per year per Center was 5 (range 2‐21) and the median period of accrual was 4 years (range 2‐8). We also evaluated the number of patients accrued and the period of accrual for each Center and all Centers but three were within the global median. Demographic and clinical characteristics of the study population are listed in Table 1. Most subjects had diffuse large cell lymphoma (DLBCL), 11 follicular lymphoma, 23 indolent lymphoma other than follicular, 15 mantle cell lymphoma, and 5 T‐cell lymphoma. The stage IV was the most represented. Based on IPI classification, more than half of the patients were at intermediate risk. Median LVEF at diagnosis was 60 (range 20‐82) for the whole group, and 58 (range 25‐80) for patients with history of cardiac diseases. Median follow‐up from the diagnosis was 42 months (range 1‐187).

**TABLE 1:** Demographic and clinical characteristics of the study population

Variable	All patients
*Demographics*	
Age, y (median)	74 (range 26‐92)
Gender %F (N)	47.7 (442/926)
	
*Clinical features*	
Histology, %DLBCL (N)	94.3 (892/946)
Stage, % (N)	
I	12.2 (115/942)
II	20.1 (189/942)
III	21.6 (203/942)
IV	46.2 (435/942)
Symptoms, %yes (N)	19.1 (174/913)
IPI, % (N)	
Low (0‐1)	20.2 (185/917)
Intermediate (2‐3)	62.6 (574/917)
High (4‐5)	17.2 (158/917)
LVEF ≥50, % (N)	86.6 (722/834)
Previous cardiac disease	32.3 (306/946)
	
*Treatment*	
R‐COMP cycles >3, % (N)	89.7 (849/946)
Radiotherapy, %yes (N)	18.9 (179/946)
Concomitant therapy	
Cardioprotective drugs only, % (N)	22.1 (209/946)
Cardiotoxic drugs only, % (N)	1.8 (17/945)
Both	0.2 (2/945)
Previous anthracycline chemotherapy	2.0 (19/946)

Abbreviations: DLBCL, diffuse large B cell lymphoma; IPI, international prognostic index; LVEF, left ventricular ejection fraction.

Reasons for treatment with nonpegylated liposomal doxorubicin (NPLD) were older age (49.2%, N = 466), pre‐existing cardiac comorbidity (31.5%, N = 298), uncontrolled hypertension (13.3%, N = 126), previous anthracycline therapy (2%, N = 19) and severe arrhythmia (2%, N = 19), other causes not specified (2%, N = 18).

Moreover, clinicians were asked whether they would have prescribed anthracycline also in the case of no availability of liposomal formulation. This question should provide a measure of the physicians' confidence in administering standard therapy in these selected patients. According to the physicians' answers about half of the patients (474:50.1%) were potentially considered for a standard therapy and the other patients were not considered for the use of anthracycline and clinicians would have provided alternative treatment or reduction of the anthracycline dose (49.9%).

### 3.2 Cardiac toxicity

About 5% (n = 51) of subjects developed major cardiotoxic events including heart failure (60.7%, N = 31), ischemic heart disease (31.3%, N = 16), acute heart attack (5.8%, N = 3), and acute pulmonary oedema (2.2%, N = 1): 9 before the end of the therapy, 22 within a year from the end of the therapy and 18 after a year from the end of the therapy. The time of two cardiotoxic events was unknown.

Among subjects that would have received standard anthracycline‐based therapy also in the case of no available liposomal formulation, the distribution of the cardiotoxic events was as follow: 10 heart failures, 3 ischemic heart diseases, 1 acute heart attack with no significance differences between the two groups (*P* = .704).

**FIGURE 1:**
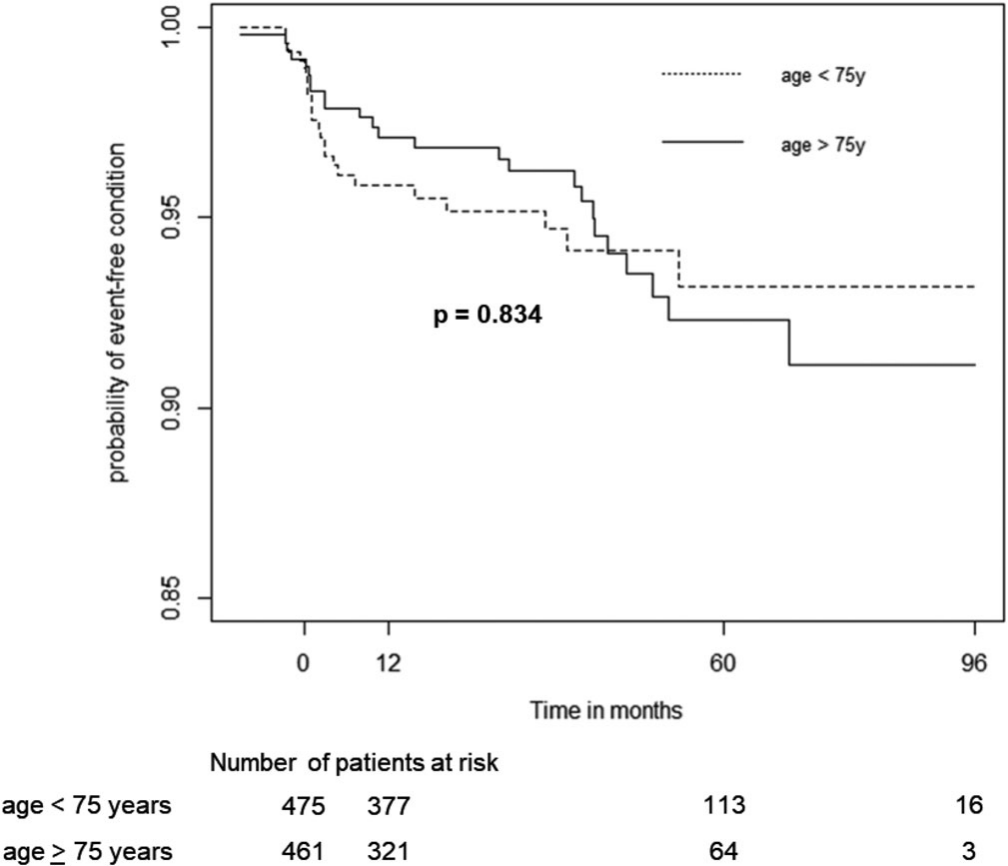
Event‐free survival curves stratified by age in patients older (solid line) or younger (dotted line) than 75 years

The overall probability of not experiencing a cardiotoxic event after 5 years was 92.6% [95% CI, 90.3%‐94.9%]. Age greater than 75 years (n = 466) did not affect the probability of event‐free condition (*χ* ^2^ = 0, *P* = .800; Figure 1).

### 3.3 Survival and causes of death

A total of 347 (36.7%) patients died from all causes including progression of lymphoma (58.8%, N = 204), cardiotoxic (5.2%, N = 18) and not cardiotoxic (11.5%, N = 40) adverse events, unknown causes (17.3%, N = 60), or second neoplasia (7.2%, N = 25).

**FIGURE 2:**
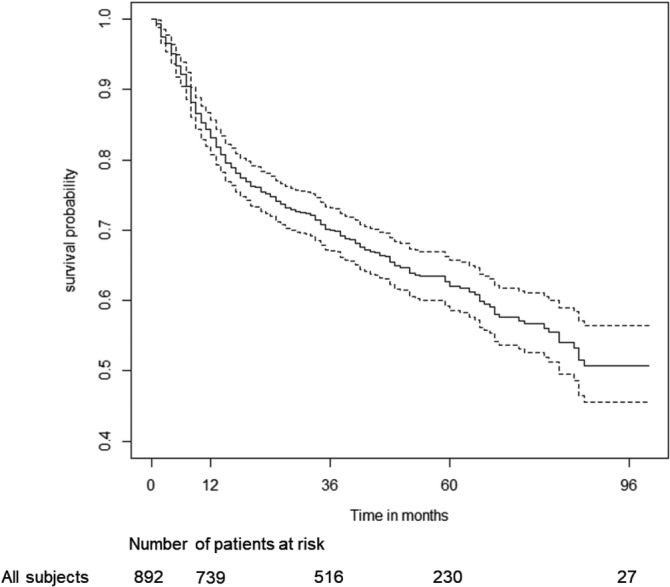
Overall survival of non‐Hodgkin lymphoma patients treated with RCOMP therapy. Dotted lines represent 95% confidence interval of Kaplan‐Meier curve

**FIGURE 3:**
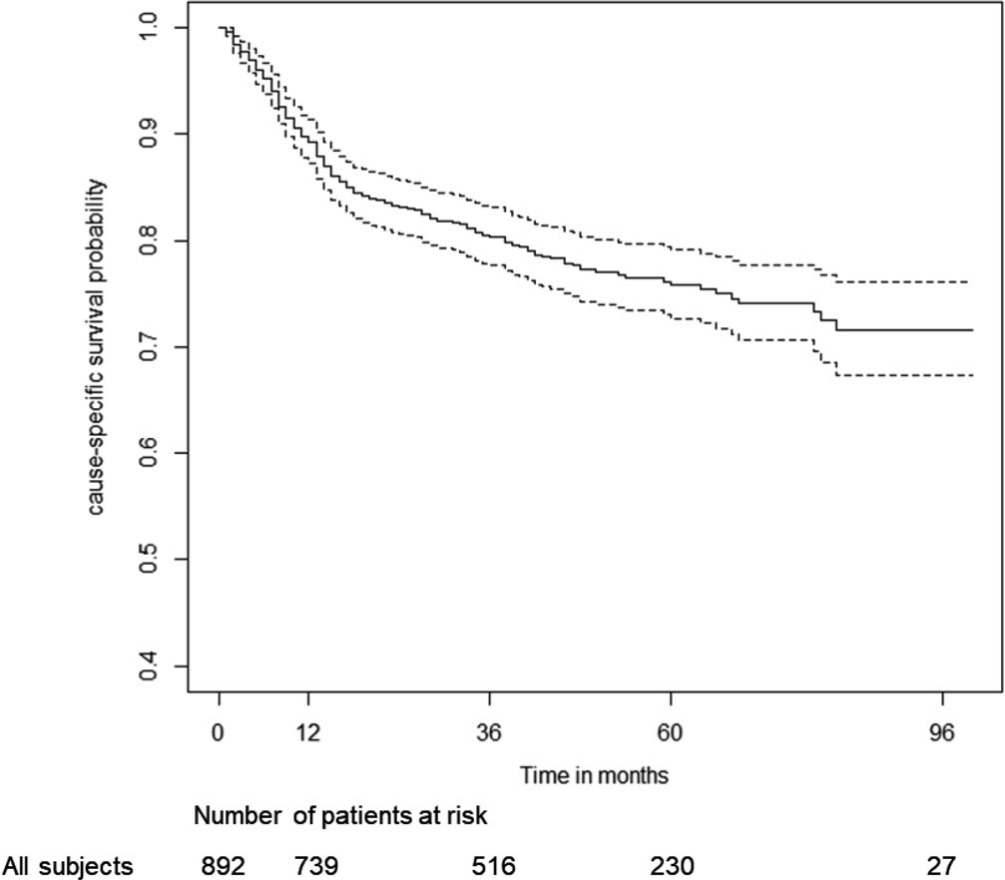
Cause‐specific survival (lymphoma‐specific survival) of non‐Hodgkin lymphoma patients under R‐COMP regimen. Dotted lines represent 95% confidence interval of Kaplan‐Meier

Considering only DLBCL the estimated 3‐ and 5‐years OS were 70.0% [95%CI: 67.0%‐73.2%] and 62.1% [95%CI: 58.6%‐65.7%], respectively (Figure 2). The estimated 3‐ and 5‐years lymphoma‐specific survival (cause‐specific survival) were 80.4% [77.7%‐83.2%] and 75.8% [72.7%‐79.1%], respectively (Figure 3).

**FIGURE 4:**
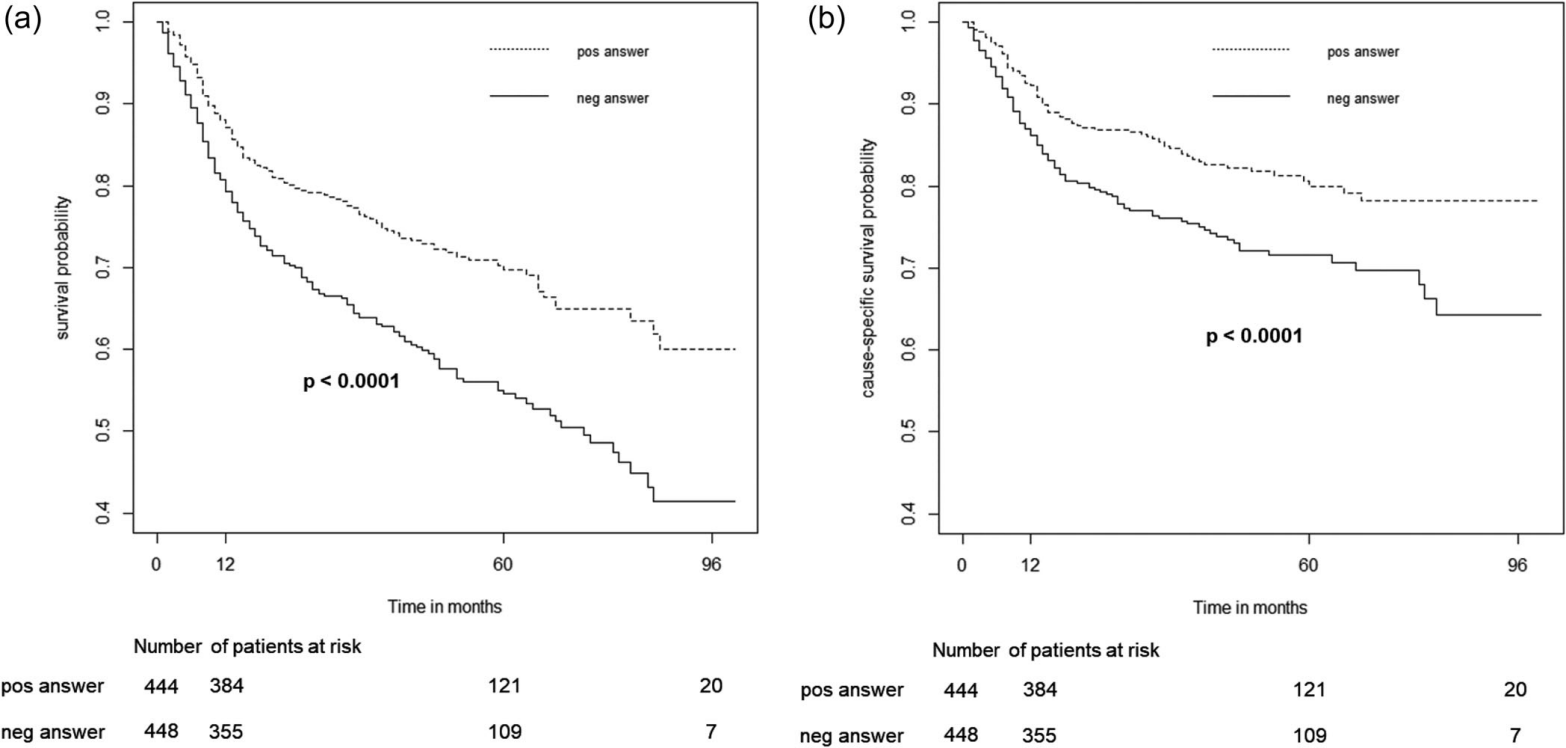
Overall (A) and cause‐specific (lymphoma‐specific) (B) survival probability stratified by clinician's opinion over the favourable (pos answer) or unfavourable (neg answer) administration of standard anthracycline‐based therapy in case of no available liposomal formulation

We did not observe any difference in OS between patients that received R‐COMP therapy because of age and patients that received the same therapy because of other causes (*χ* ^2^ = 0.0, *P* = .900). We did not observe any difference in OS either between patients with abnormal and normal LVEF (*χ* ^2^ = 2.9, *P* = .09) nor between patients with and without a previous cardiopathy (*χ* ^2^ = 1.1, *P* = .300). Subjects that according to physicians' opinion would have received standard anthracycline‐based therapy also in the case of no available liposomal formulation had both a higher overall and lymphoma‐specific survival probability than subjects that would not have received it (*χ* ^2^ = 20.7, *P* < .0001 and *χ* ^2^ = 11.0, *P* < .0001, respectively; Figure 4A,B).

On the whole, survival was negatively influenced by adverse IPI (high and intermediate vs low IPI: HR [95%CI], 4.0 [2.5‐6.2] and 2.5 [1.6‐3.7], respectively; *P* < .0001). However, an adequate number of R‐COMP cycles positively affected the survival probability also in intermediate/high IPI class of patients as shown in Table 2.

**TABLE 2:** Five‐year estimation of survival probability stratified by IPI score and number of total R‐COMP cycles

	Number of R‐COMP cycles	
5‐year survival probability, % [95% CI]	≤3	>3
Low IPI	68.4% [50.4%‐92.9%]	80.7% [73.8%‐88.3%]
Intermediate IPI	24.5% [12.0%‐50.0%]	64.9% [60.3%‐69.9%]
High IPI	NA	50.0% [41.4%‐60.3%]

Abbreviations: CI, confidence intervals; IPI, international prognostic index; NA, not available.

**FIGURE 5:**
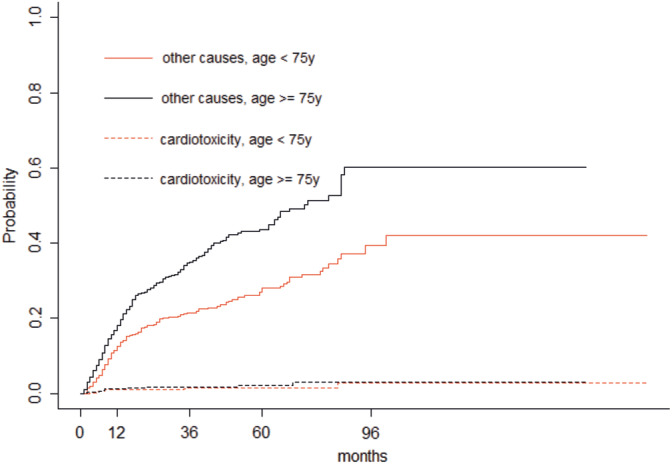
Competing risk analysis of death from cardiac events and all other causes

Among the 17 patients that died because of cardiac events, 14 were the above‐mentioned subjects that experienced a cardiac event during the observation period. The chance of dying from all other causes was significantly higher in subjects older than 75 years (*P* < .0001) whereas no difference was seen for lethal cardiac events between the two age groups (*P* = .431; Figure 5).

### 3.4 Response to therapy

Overall 5083 cycles were infused: 684 patients (72.3%) received 6 or more cycles (max. 8), 165 (17.4%) received 4 or 5 cycles and 97 patients (10.2%) less or equal to 3 cycles.

ORR was attained in 85.2% (806) of subjects with CR in 72.6% (N = 687) and PR in 12.6% (119), respectively, whereas a few patients did not respond to therapy (about 14% = 134) or were withdrawn because of premature death (0.6% = 6). Responses in the 306 patients with previous cardiopathy were CR 73.5% (N = 225), PR 10.1% (N = 31) and PD 16.3% (N = 50). Complete remission rate was superior at the limit of significance (*P* = .01) for patients who would have been able to use standard doxorubicin (76%) vs patients who would not have used doxorubicin (69%).

As expected, adverse prognosis negatively affected both PR (intermediate IPI odds ratio (OR): 2.7 [95% CI 2.0‐3.7], *P* < .0001; high IPI OR: 2.6 [95% CI 2.1‐3.3], *P* < .0001) and NR (intermediate IPI OR: 5.6 [95% CI 4.3‐7.2]; high IPI OR: 16.4 [95% CI 13.0‐20.8), *P* < .0001] whereas symptoms and consumption of cardioprotective drugs significantly changed only the likelihood of NR (OR [95% CI]: 1.8 [1.1‐2.9], *P* = .019; 0.5 [0.3‐0.9], *P* = .020, respectively).

**FIGURE 6:**
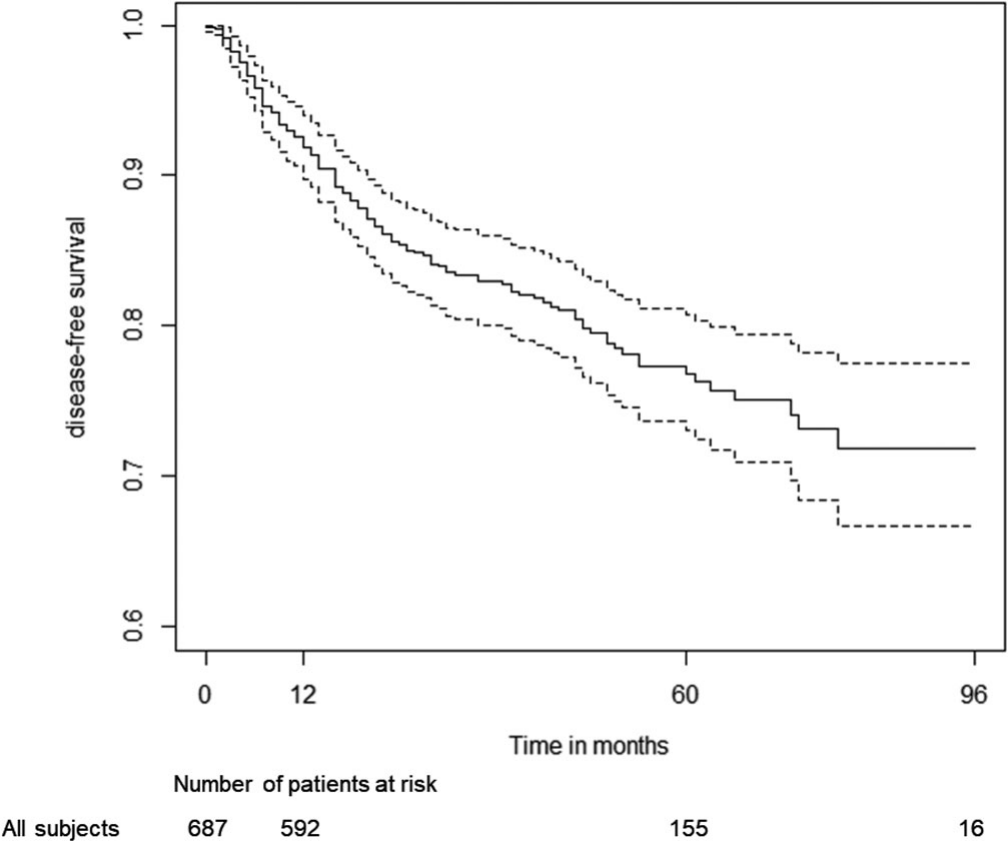
Estimated probability of not experience relapse (disease‐free survival). Dotted lines represent 95% confidence intervals of Kaplan‐Meier curve

During follow‐up, 19.4% (N = 133) of the patients that successfully responded to R‐COMP regimen relapsed. The disease‐free survival probability after 5 years of observation was 76.8% (95% CI: 73.0%‐80.7%) (Figure 6). The chance of relapse was comparable between patients with abnormal and normal LVEF (*χ* ^2^ = 1.3, *P* = .300) at diagnosis.

When compared to patients with better prognosis, patients with high IPI showed 2.9 (95%CI: 1.5‐5.5, *P* = .002) greater risk of relapse while patients with intermediate IPI showed an increased but not statistically significant risk of relapse (HR = 1.6 [95% CI: 1.0‐2.8], *P* = .074).

## 4 DISCUSSION

The CHOP scheme was ideated in 1972 and continues nowadays to be considered the standard treatment for DLBCL. Different attempts have been made over the years to improve the results of CHOP regimen^22^, ^23^, ^24^, ^25^, ^26^ but the only real improvement in CHOP results was the introduction of a monoclonal anti‐CD20 antibody, rituximab, in combination. The study by Coiffier et al^27^ for the first time after several years showed a 20% improvement in OS. The exceptional effectiveness of the scheme is unfortunately burdened by a risk, contained but present, of cardiotoxicity due to the presence of anthracycline. Heart failure induced by anthracyclines is unfortunately unpredictable, but it is well‐known that the accumulation of the drug induces a left ventricular dysfunction particularly over a cumulative dose of 450 mg. Other risk factors include young age at diagnosis, female sex, chest radiation, and presence of cardiovascular risk factors (diabetes, hypertension, dyslipidemia, obesity, and smoking). In a recent large study on 2440 patients^28^ treated with anthracyclines the cumulative 5‐year risk of cardiac heart failure (CHF) with all‐cause mortality as competing risk was 4.6% after 3 to 5 cycles of R‐CHOP, 4.5% after 6 and 7.9% after more than 6 cycles. Cumulative 5‐year risk for patients treated without anthracyclines was 0.8%. Moreover, using anthracyclines in first line lymphoma therapy increases the risk of CHF in patients without a previous history of heart disease.

In a large retrospective study by Maraldo et al,^29^ 1238 first cardiovascular events were recorded in 703 patients. The factors resulting in significant predictors of cardiovascular disease were the mean heart radiation dose per 1 Gy increase (HR 1.015 [95% CI 1.006‐1.024] *P*:.0014) and the dose of anthracyclines per 50 mg/m^2^ increase in cumulative dose (HR 1.077 [95% CI 1.021‐1.137] *P*: .0064). An interpatient variability in risk of anthracyclines induced CHF confirms a genetic association as reported in several studies.^30^, ^31^, ^32^, ^33^, ^34^ As reported in the literature the risk of cardiotoxicity is significant in patients without risk factors therefore patients with signs of cardiac failure are preventively excluded from treatment with R‐CHOP. The attempt to use anthracyclines free regimen has not led to expected results.^35^, ^36^

For these reasons there is a strong desire to consider alternative strategies if anthracyclines cannot be avoided: (a) less cardiotoxic analogs for example in adults liposomal‐encapsulated doxorubicin, (b) longer anthracyclines infusion duration for example an infusion of at least 6 hours has been shown to be cardioprotective,^37^ (c) cardioprotective agents like dexrazoxane, and (d) reduction of anthracycline dosage, as in mini‐CHOP regimen.^38^

Dexrazoxane is the only one drug approved in US as cardioprotective agent^39^, ^40^ but its use is limited to a small percentage of patients due to an unjustified concern that this drug may interfere with antitumor activity and increase the risk of second neoplasms.^41^, ^42^, ^43^ The liposomal formulation showed in large randomized controlled trials and meta‐analysis on metastatic breast cancer a reduction of the cardiotoxic risk, observed with conventional anthracyclines without affecting antitumor activity.^44^, ^45^, ^46^

The safe cardiologic profile of liposomal anthracyclines was well‐documented by studies in which this drug was administered in selected patients at very high risk of cardiotoxicity (advanced age, anthracyclines pretreatment, and concomitant cordiotoxic drug).^47^, ^48^, ^49^, ^50^

The aim of our study was to evaluate in a multicentric and real‐life clinical setting the impact of NPLD, as replacement for conventional doxorubicin in R‐CHOP regimen, on efficacy and safety within high or very high‐risk population suffering from lymphoma. The main reason for using NPLD was age; that is why the median age was quite high (74 years) with a substantial part of the population over 79 years (83 patients: 9%). One third of the patients were treated with NPLD because of an history of cardiopathy except uncontrolled hypertension observed in 13% of patients. The population we studied is strongly negatively selected and according to the clinicians' declaration about half of the patients would not have been treated with doxorubicin implying the risk of undertreatment.^51^

The incidence of cardiac complications was 5.3% in the whole analyzed population, heart failure was the most frequent toxic effect and was reported in 31 patients (3.2%), according to literature where the incidence of heart failure in patients treated with standard doxorubicin was roughly 2% to 3%.^52^, ^53^, ^54^

Therefore, despite a high risk selected population the incidence of cardiac toxicity was mild and superimposable with those reported in populations treated with standard doxorubicin. The NPLD can be used in cardiopathic patients without increasing the cardiac toxicity, meaning that we can treat more patients with the best treatment available. It is very interesting to observe that physicians should not have used standard doxorubicin in about half of the referred patients; the bad prognosis of this group of patients was confirmed by the fact that although they were treated with an optimal regimen they achieved worse results than patients treated with standard anthracyclines. However, the use of NPLD permits us to treat patients that, with high probability, would have been treated with alternative schemes, certainly less effective than R‐CHOP particularly in DLBCL.

At the same time, we have treated a group of patients considered potentially available for standard doxorubicin obtaining very high complete remission rate confirming that the use of NPLD did not reduce the efficacy of the treatment. The first objective of the study was to confirm the safety, particularly cardiac, of this NPLD even in a group of patients with high‐risk situations due to age and/or pre‐existing cardiopathy. We observed 73% of complete remission that is completely superimposable with the results reported in other studies using R‐CHOP particularly if we consider the high median age of the population treated as reported in the randomized phase III study by Fridrik et al.^17^ The overall survival after a median observation of 34 months (range 1‐187 month) was 70% (95% C.I.: 67.4‐73.4%) and the DFS after a median observation of 24 months was 76% (95% C.I.: 74‐80.7%).

In conclusion, using this liposomal formulation on a high or very high‐risk population, the incidence of cardiotoxicity was absolutely comparable with the events historically reported using standard doxorubicin. The use of NPLD that significantly reduces the accumulation and consequently the toxic effect of doxorubicin on myocites permits to use the standard treatment in a large proportion of the population and the more patients we can treat adequately the more patients we can cure. Our study confirms also the effectiveness in term of complete remission rate of the COMP regimen. Finally we must consider the possible bias of selection due to the retrospective nature of the study and for this reason our conclusions should be cautious but we are confident that either the evaluation performed by a single center or the large number of patients recruited can mitigate this potential bias.

## CONFLICT OF INTEREST

The authors have no competing interest.
